# Open fracture dislocation of the talus with nearly total extrusion and with tri-malleolar fracture: A case report and literature review

**DOI:** 10.1097/MD.0000000000043827

**Published:** 2025-08-15

**Authors:** Yu-Jui Chang, Chi-Hsiang Hsu, Yuan-Hao Yen, Jen-Hung Chen, Shan-Ling Hsu

**Affiliations:** a Department of Orthopaedic Surgery, Kaohsiung Chang Gung Memorial Hospital and Chang Gung University College of Medicine, Kaohsiung, Taiwan; b Division of Plastic and Reconstructive Surgery, Department of Surgery, Kaohsiung Chang Gung Memorial Hospital and Chang Gung University College of Medicine, Kaohsiung, Taiwan.

**Keywords:** associated fracture, avascular necrosis, open dislocation, open talus extrusion with tri-malleolar fracture, reimplantation, talus extrusion

## Abstract

**Rationale:**

Open talus fracture with extrusion is a rare condition of trauma. We report a 5-year follow-up of an open talus fracture with extrusion associated with a tri-malleolar fracture that was successfully treated with reimplantation and fixation.

**Patient concerns:**

We report the case of a 73-year-old female with a diagnosis of left foot open talus fracture with extrusion. She also presented with an ipsilateral ankle tri-malleolar fracture.

**Diagnoses:**

Open talus fracture with extrusion and associated with a tri-malleolar fracture.

**Interventions:**

Surgery with reimplantation and fixation.

**Outcomes:**

The extruded talus was reduced, and the fracture healed approximately 9 months after the operation. There were no adverse effects at the 5-year follow-up point. The patient showed excellent performance, and no obvious sequelae were found.

**Lessons:**

To the best of our knowledge, this is the first report of an open talus fracture with extrusion associated with a tri-malleolar fracture.

## 1. Introduction

Fractures of the talus are relatively uncommon in clinical practice, especially when dislocation and even extrusion occur simultaneously. Based on the literature review,^[1,2]^ open fracture with extrusion of the talus have a high possibility of complications, such as avascular necrosis (AVN) of the talus or infection and could have a variable prognosis. However, early intervention with meticulous debridement, completely anatomic reduction, and appropriate postoperative protection with rehabilitation are the keys to successful treatment.

We report a case with open talus fracture dislocation with nearly total extrusion. We adopted the reimplantation surgery as the treatment. The fracture achieved bony union at 6 months after the surgery, and the patient returned to her daily life, and no obvious complications were observed at the 5-year follow-up point. Our patient was informed that the data concerning the case would be submitted for publication.

## 2. Case presentation

A previously healthy 73-year-old female pedestrian was bumped by a car at a speed of approximately 70 km/h while riding her bicycle on an asphalt road. She was brought to our institution within 2 hours of the accident. After a general survey, her injuries involved only the musculoskeletal system. The left foot was noted to be in a position of supination with respect to the leg and was flaccid. There was a 5-cm, longitudinal-type open wound over the lateral side of the foot with the extruded talus and no apparent loss of the soft tissue. The neurovascular status of the lower extremity was completely intact. Inspection of the talus revealed much dirty debris spotted and fracture of the body.

**Figure 1.**
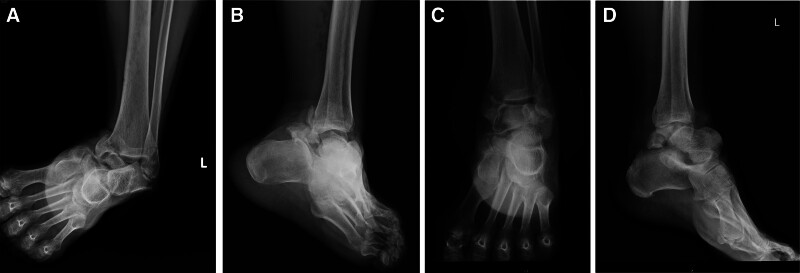
AP and lateral radiograph of the left ankle demonstrating fracture and dislocation of the talus with tri-malleolar fracture (A, B) and after reduction (C, D).

**Figure 2.**
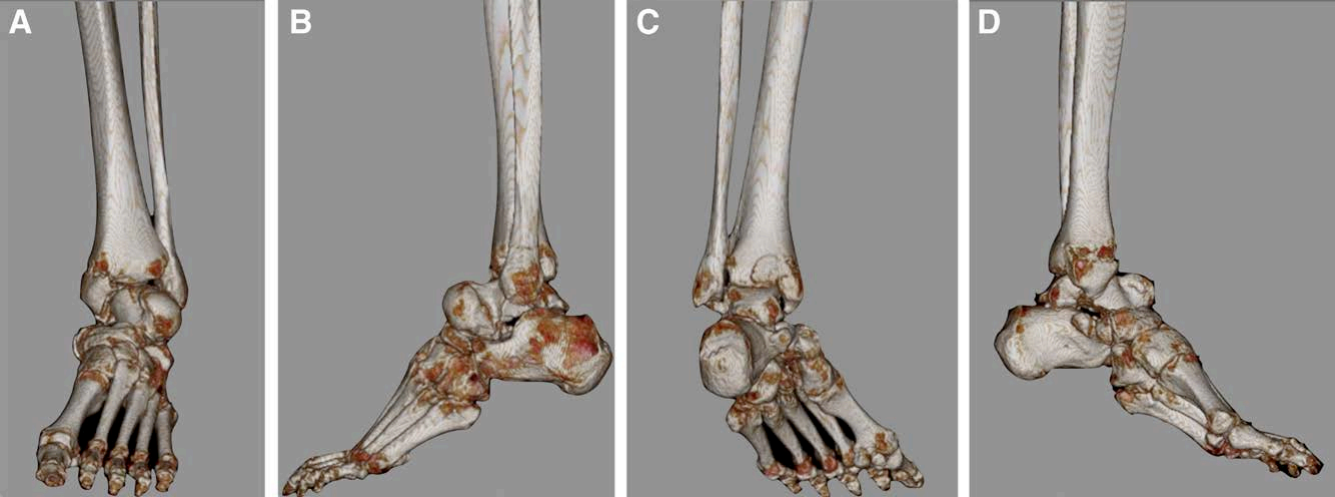
Musculoskeletal computed tomography (CT) scan with three-dimensional reconstruction of the left foot and ankle showing pan-talar dislocation (subtalar, talonavicular, and tibiotalar joint) and body fracture; and displaced medial malleolar fragment with disrupted medial side mortise, the posterior malleolar fragment involved <25% of the articular surface with <2 mm displacement, and the lateral malleolar Danis–Weber type B fracture with a congruent lateral side mortise.

Radiographs of the left foot and ankle (Fig. 1) revealed a talus fracture and dislocation with nearly total extrusion and a tri-malleolar fracture. Considering the aforementioned factors, including wound size, neurovascular status, soft tissue characteristics, and radiographic finding, the injury was diagnosed as a Gustilo–Anderson type II open talus fracture and dislocation, and associated with a left ankle tri-malleolar closed fracture. Then we performed urgent reduction of the dislocated talus at the emergency department. Left ankle and foot computed tomography (CT) scan with three-dimensional reconstruction (Fig. 2) was arranged and revealed that displaced medial malleolar fragment with disrupted medial side mortise, and the posterior malleolar fragment involved <25% of the articular surface with <2 mm displacement and maintained syndesmotic stability, while the lateral malleolar fracture was located at the level of the syndesmosis with a congruent lateral side mortise, classified as Danis–Weber type B. Then the surgical plan and its associated risks were explained to the patient in detail prior to surgery, and informed consent was obtained in accordance with standard surgical ethics and guidelines.

### 2.1. Initial surgery

**Figure 3.**
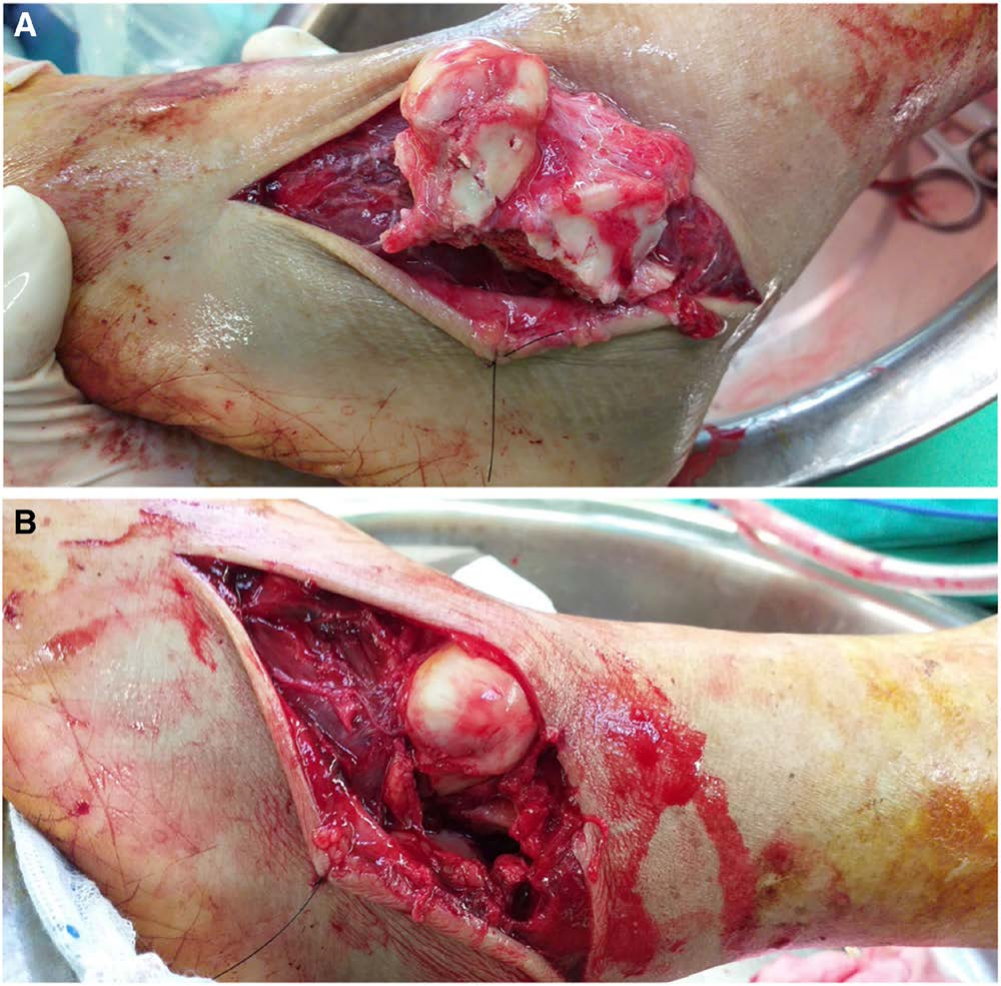
Intraoperative representative images showing the extruded talus (A, B).

The patient was taken to the operation room 3 hours after arrival at our emergency department. Emergent debridement with pulsatile normal saline lavage, and thorough debridement were performed via curettage for the talus, which was then gently reimplanted into its anatomical position under fluoroscopic guidance with respect to the tibia and subtalar joints and the additional anteromedial approach of the talus was made to facilitate exposure and reduction (Fig. 3). Then immediate definite open reduction and internal fixation for talus fracture with paired retrograde 3.5-mm cancellous screws and temporary fixation for medial malleolar fracture with two 1.8-mm Kirschner-wires were performed within 6 hours after the injury. We also performed primary repair for the residual ruptured ankle ligaments and wound.

**Figure 4.**
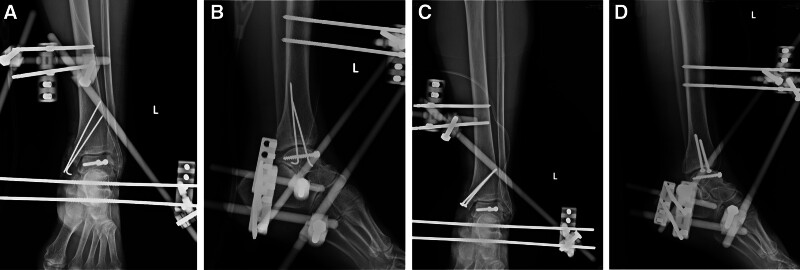
Postoperative follow-up radiograph after the initial operation showing the talus after reimplantation and external fixation (A, B) and after the second stage operation (C, D).

Given the extensive soft-tissue injury and risk of ankle instability, a standard triangular ankle-spanning external fixator was applied with two 4.5-mm Schanz pins in the tibial diaphysis and two 4.5-mm Schanz pins in the calcaneus, connected with rods for the enhancement of gross ankle stability including posterior and lateral malleolar fractures and for the assistance of soft tissue healing (Fig. 4). The ankle was maintained in a neutral position. All hardware positions and the fracture reduction were confirmed with fluoroscopy.

### 2.2. Staged surgery

No pathogens were detected according to the surgical wound culture report. The second stage was performed 6 days after the initial surgery. We changed the Kirschner-wires to 3.5-mm cancellous screws for the definitive fixation for medial malleolar fracture. Regarding the posterior and lateral malleolar fractures, under the consideration of preoperative X-rays and CT scan revealed minimal displacement of both fragments and intraoperative maintained syndesmosis stability with a congruent and stress-stable mortise and considered that the external fixator could also provide sufficient mechanical support, we decided not to perform further fixation such as plate or screw for both fragments. We performed debridement of the soft tissue around the talus and the talus was not dislocated during this procedure. The goal was to preserve soft tissue integrity and the blood supply to the talus and adopt a strategy involving the use of fewer implants to achieve acceptable stability.

### 2.3. Antibiotics

Intravenous cephalosporin and gentamicin were initiated preoperatively and were continued at a dosage of 1 g 3 times daily for 7 days, followed by cephalosporin for 3 weeks and then a shift to the oral form after discharge. The external fixator for the ankle and foot was left in place for approximately 6 weeks.

### 2.4. Soft tissue management

**Figure 5.**
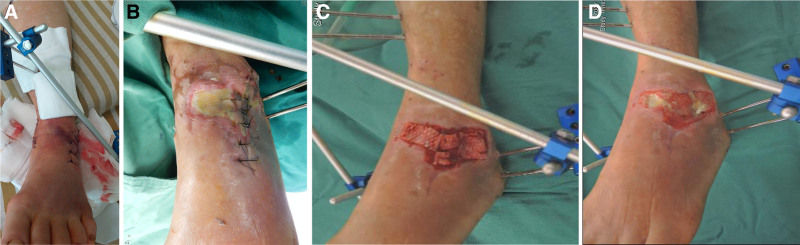
Photographs of the left ankle showing after mild bullae formation surgery. No infection sign was seen.

However, poor wound healing was noted after the surgery (Fig. 5), and a plastic surgeon was consulted for further management. There was skin necrosis over the anterior to lateral aspect of the left ankle. After serial debridement and wound bed preparation for 19 days, a 10 × 3.5 cm wound with a full layer defect was generated, with exposure of the extensor tendons, and the anterior talofibular ligament.

After providing informed consent, the patient preferred reconstruction with artificial material, rather than a local flap or free flap. We proceeded with surgery of artificial dermis reconstruction with fenestrated Terudermis (Terumo Corp., Tokyo, Japan), and applied negative pressure wound therapy (NPWT), vacuum-assisted closure (KCI Inc., San Antonio, TX), at a pressure of 125 mm Hg in continuous mode. The vacuum-assisted closure was removed 7 days after surgery, and wound care with a foam dressing was continued once per day. The silicon layer of the Terudermis was removed after incorporation of the neo-dermis, 19 days after surgery. Without further skin grafting surgery, the wound completely healed within 43 days.

### 2.5. Prognosis

After management, all the wounds healed well and there was no infection (Fig. 5). Ambulation under a walker with partial weight-bearing began at the eighth postoperative week and then progressed to full weight-bearing without assistance at the ninth month.

**Figure 6.**
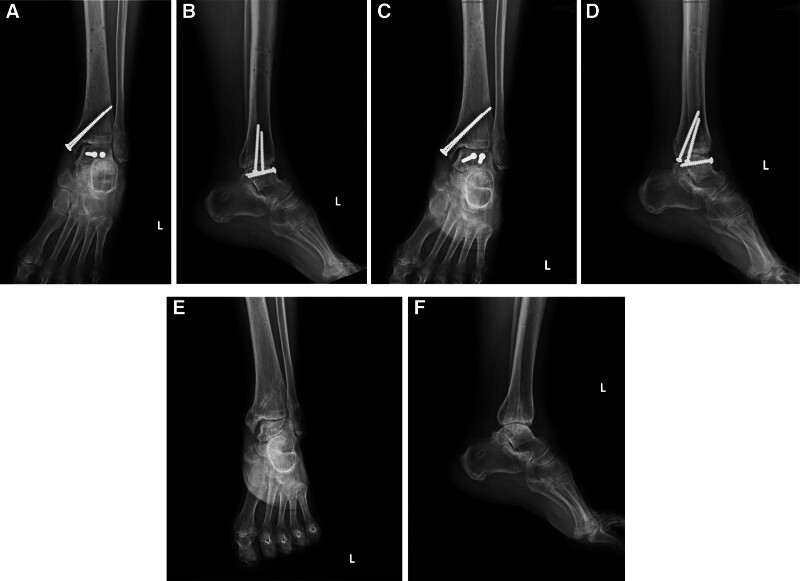
AP (A) and lateral (B) radiographs obtained 3 months after surgery demonstrating callus formation; radiographs showed bony union at 6 months after surgery (C, D); and after implant removal at 2 years after surgery (E, F).

**Figure 7.**
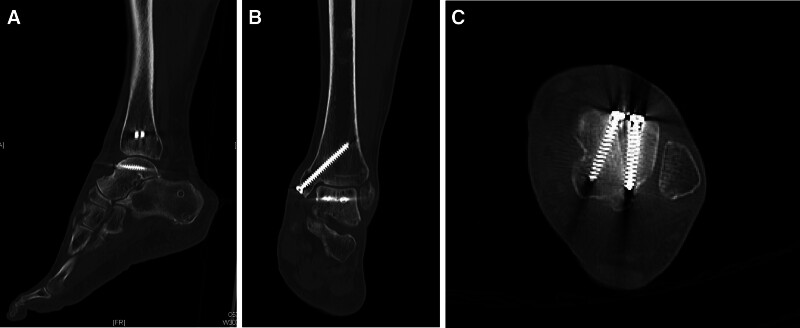
Follow-up CT with three-dimensional reconstruction of left foot obtained 10 months after surgery, revealing fracture site bony union and no post-traumatic osteoarthritis.

Follow-up plain film of the left foot from the out-patient department revealed fracture-site healing with callus formation after approximately 3 months (Fig. 6). After 6 months of surgery, the patient’s talus and malleoli had achieved bony union (Fig. 6), and she could ambulate with only 1 cane under full weight-bearing, while no serious complications, such as necrosis or collapse were observed (Fig. 6). Under the consideration of the implant irritation, and it may have influence of ankle range of motion, especially dorsiflexion, the screws for talus fixation were removed after 2 years of surgery (Fig. 6). A follow-up left ankle CT scan (Fig. 7) revealed good mortise joints, and no post-traumatic arthritis was observed.

**Figure 8.**
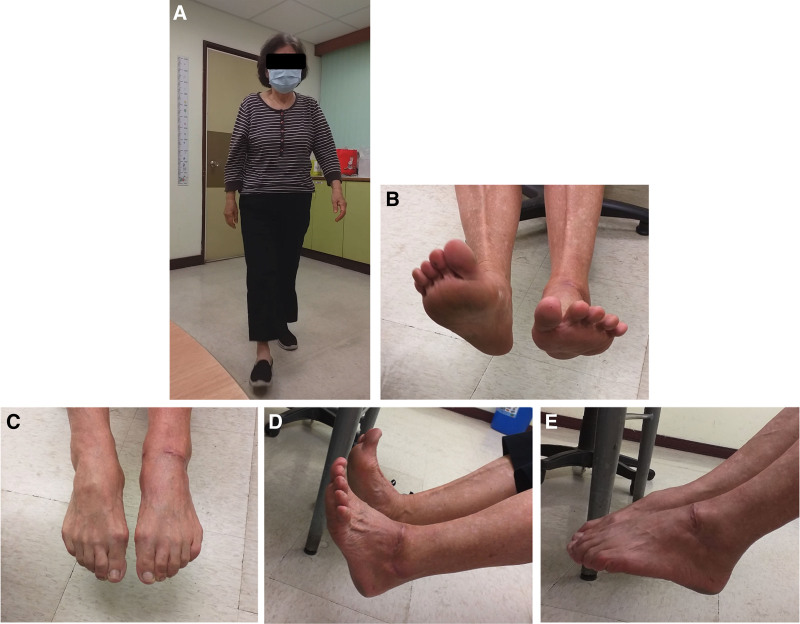
Nearly normal appearance of the involved limb on this patient after 3 years follow-up (A); mild limitation of range of motion was noted (B–E) but the gait was stable and had no impact on her daily life.

**Figure 9.**
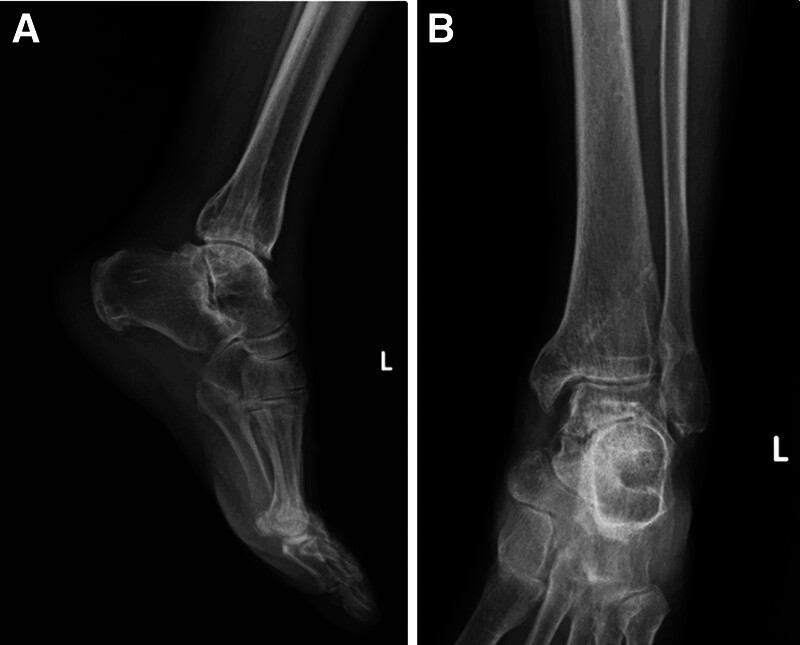
AP (A) and lateral (B) radiographs obtained 5 years after surgery demonstrated acceptable mortise of the left ankle joint.

At the 3-year follow-up after the injury, there was no leg discrepancy, and the patient’s gait was stable without any limp, and with no obvious impact on her daily life. The postoperative American Orthopedic Foot and Ankle Society (AFOAS)^[3]^ at 9 months to 5 years ranged from 63 to 98 (Table 1). She did not complain of any giving way or instability in the ankle. She was active in bicycling and hiking, and she tolerated these activities well, but occasional soreness was still noted. Physical examination of the left ankle and foot revealed that the degree of dorsiflexion was limited, while others were free (Fig. 8). The range of motion of the ankle was pain-free, with plantar flexion of 35° and dorsiflexion of 10° compared to the uninjured side with plantar flexion of 35° and dorsiflexion of 25° (Fig. 8 and Table 1). No instability of the injured ankle or subtalar joints was noted. Furthermore, no obvious complications such as recurrent infection, avascular necrosis of the talus, or tibiotalar joint collapse, were noted after 5 years of follow-up (Fig. 9).

**Table 1:** Progression of American Orthopaedic Foot and Ankle Society (AOFAS) scores and ankle range of motion over a 5-year follow-up period.

Variable/time	9 mo	1 yr	2 yr	3 yr	4 yr	5 yr
Dorsiflexion (°)	5	5	5	10	10	10
Plantarflexion (°)	25	30	30	35	35	35
Pain	30	30	30	40	40	40
Function	23	38	44	48	48	48
Alignment	10	10	10	10	10	10
AOFAS score	63	78	84	98	98	98

## 3. Discussion

### 3.1. Anatomy and blood supply

The talus is known for its unique blood supply which poses a threat to its vascular integrity at the time of injury. Historically, total talar dislocation has been managed by primary talectomy and tibiocalcaneal arthrodesis. Extrusion of the talus may compromise the blood supply and eventually lead to avascular necrosis, especially circumstances without any remaining soft-tissue connections. Surgical strategies, including early debridement, early reimplantation with anatomic reduction, and rigid fixation of the talus could provide reliable clinical outcomes and decrease complications such as infection or avascular necrosis on the basis of our case and literature review.^[1,2]^

### 3.2. Injury mechanism

Open talus dislocation with extrusion is a rare traumatic injury according to the literature review.

The term aviator’s astragalus was used to describe this type of injury.^[4]^ The injury mechanism^[5]^ involves excessive dorsiflexion with disruption of the subtalar, tibiotalar, and talonavicular joints. Total dislocation of the talus results either from excessive supination or from excessive pronation; accordingly, the dislocated talus lies either laterally or medially. A first-degree supination injury results in medial subtalar dislocation; a second-degree supination injury results in medial subtalar dislocation with talocrural subluxation; and a third-degree supination injury results in total lateral dislocation of the talus. A first-degree pronation injury results in lateral subtalar dislocation; a second-degree pronation injury results in lateral subtalar dislocation, together with talocrural subluxation; and a third-degree pronation injury results in total medial dislocation of the talus.

### 3.3. Soft tissue protection and wound management

Traditionally, large or heavily contaminated defects are managed with local rotational flaps or free tissue transfer to achieve reliable coverage and promote healing.^[6]^ Flap reconstructive surgery represents the gold standard method, for wounds with tendon and bone exposure. However, flap procedures carry risks of donor-site morbidity, increased operative time, and higher perioperative complexity. Nowadays, artificial dermis has been proven valuable for trauma and oncological reconstruction, as an alternative option for patients who are not suitable for flap surgery. Most types of artificial dermis require second-stage skin grafting after a 2- to 4-week period of tissue incorporation,^[7–10]^ except for AlloDerm, which can be applied in single-stage operations with simultaneous skin grafting.^[11,12]^ However, this product is not available in our country.

Terudermis, is an artificial dermis composed of a collagen sponge reconstituted from heat-denatured bovine dermal type 1 collagen that is cross-linked via a dehydro-thermal process. Compared with other artificial dermis products, it is characterized by an equipped silicone membrane layer, to prevent infection and control moisture flux. Terudermis has been shown to be noninferior to other dermal substitutes in animal models.^[13]^

NPWT has been reported to accelerate the tissue incorporation of artificial dermis, such as AlloDerm,^[11]^ Integra,^[14]^ Terudermis, and Pelnac,^[15]^ over wounds. Hsu et al used an NPWT dressing on the fenestrated Terudermis to increase the rate and success of revascularization over the wounds with tendon and bone exposure, with an 87% success rate in patients with acute trauma.^[7]^ For our patient, the wound presented with tendon and ligament exposure and was treated with fenestrated Terudermis and 1-week NPWT dressing. There was no further skin grafting surgery and eventually, the wound was completely healed.

Previous studies^[16]^ have reported that favorable outcomes with artificial dermis and NPWT for moderate soft-tissue defects, particularly when contamination is controlled and wound margins are viable. While flap coverage remains the gold standard for larger or nonviable wounds, our result demonstrates that Terudermis and NPWT can be a viable alternative in selected cases, offering good coverage with less donor-site morbidity and acceptable functional outcomes, supporting its use as a feasible alternative in carefully selected patients.

### 3.4. External fixation

External fixation plays an important role in the management of severe open talus injuries with extrusion, especially when combined with associated ankle fractures. Previous studies^[2,17]^ have emphasized that external fixators can provide provisional or definitive stabilization in the setting of massive soft-tissue disruption, protect the tenuous reimplanted talus, and allow staged soft-tissue management or flap coverage. In our case, the spanning fixator helped maintain hindfoot alignment, reduced micromotion across the tibiotalar and subtalar joints, and minimized additional vascular compromise. Application of external fixation is consistent with damage control principles for high-energy ankle and foot trauma and should be an integral part of the initial management.^[18]^

### 3.5. Treatment options

The treatment of talar extrusion is composed of 2 stages: first, meticulous debridement to avoid short-term complications especially infection. The second phase involves reconstructing the talus, and options include talar preservation, replacement of the talus, and tibiocalcaneal arthrodesis.^[19]^

Several reports have recommended reimplantation of the talus in cases of total extrusion. Kaeleen et al^[2]^ reported 14 cases of open pan-talar dislocation and successfully managed with urgent surgical debridement and reimplantation of the talus after a mean follow-up of 45.1 months. Assal et al^[18]^ also recommended the reimplantation of a totally extruded talus, noting that the risk of osteonecrosis or infection related to the loss of vascularity is relatively low. Except for the severe contamination of the extruded talus, reimplantation should be the priority in the management of these cases. Smith et al^[1]^ reported that 19 patients with the extruded talus underwent reimplantation and that only 2 patients had infections; supporting the salvage of the extruded talus was relatively safe and thus reimplantation should be strongly considered. Overall, the prognosis is variable and depends largely on the associated fracture pattern. Based on a review of the literature,^[1,20,21]^ reimplantation of the talus is a valid curative option that can achieve satisfactory outcomes. In our case, we adopted the reimplantation strategy combined with internal fixation and yielded an acceptable mid-term outcome.

Total talar prosthesis replacement after talar extrusion is also a useful method for treating these patients. Ruatti et al^[22]^ performed total talar prosthesis replacement after talar extrusion in a 51-year-old patient with encouraging results after a 2-year follow-up period. However, much longer follow-up is necessary. Arthrodesis could be adopted as a salvage method if reimplantations failed. In a previous report,^[23]^ Ortiz-Cruz et al used tibiocalcaneal arthrodesis after talectomy because preservation of the talus was unfeasible. Otherwise, Huang et al^[24]^ used an antibiotic cement spacer and staged tibiocalcaneal fusion with a femoral head allograft with good clinical outcomes after a 2-year follow-up. In brief, the strategy is still controversial nowadays. Treatment depends on the presence of infection and the possibility of preservation of the talus.

### 3.6. Complications

Complications can be divided into 2 categories: short-term (infection) and long-term complications, such as collapse, AVN, and post-traumatic arthritis. With respect to our case, there were no adverse effects or complications noted after reimplantation for a 5-year period, except for mild dorsiflexion limitation (Fig. 8). There is no need for local injection or other analgesics for this purpose currently. Although AVN is a well-documented and common complication in extruded talus injuries, certain factors may have contributed to the preservation of talar vascularity in this case. Early and meticulous debridement, prompt anatomic reduction, and rigid internal fixation likely helped minimize additional vascular compromise. Furthermore, residual soft-tissue attachments, even if limited, may have provided sufficient revascularization potential. Similar isolated reports^[1,2,25]^ have highlighted that immediate reimplantation and careful handling of soft tissues can reduce the risk of AVN, despite the severity of extrusion. Otherwise, punctate bleeding was observed from the fracture surfaces of the talar neck and body Intraoperatively, indicating that some vascular supply was maintained despite the extrusion. This finding has been recognized as a favorable prognostic factor for talar viability and may partly explain the absence of avascular necrosis in this case.^[26,27]^

In addition, although the patient’s relatively older age may have contributed to less soft-tissue healing and less revascularization potential, the satisfactory mid-term functional outcome benefit from the effectiveness of early debridement, stable fixation, and meticulous soft-tissue management. However, further follow-up is needed to confirm the long-term viability of the talus and functional recovery of the patient in such rare scenarios.

## 4. Conclusions

Open fracture and dislocation of the talus with extrusion require surgical intervention, and complications such as post-traumatic arthritis or avascular necrosis are common, but the need for revision surgery is not frequent. Here, we present a case with an acceptable clinical outcome. The reimplantation should be considered in a priority and could achieve fixation in the first stage.

## Author contributions

**Conceptualization:** Yu-Jui Chang, Chi-Hsiang Hsu.

**Data curation:** Yu-Jui Chang, Yuan-Hao Yen, Jen-Hung Chen.

**Investigation:** Yu-Jui Chang.

**Methodology:** Yu-Jui Chang.

**Project administration:** Yu-Jui Chang.

**Supervision:** Chi-Hsiang Hsu, Jen-Hung Chen.

**Writing – original draft:** Yu-Jui Chang, Yuan-Hao Yen.

**Writing – review & editing:** Chi-Hsiang Hsu, Shan-Ling Hsu.
